# Evaluation of the safety and efficacy of ^225^Ac-PSMA-CY313 in metastatic castration-resistant prostate cancer: preliminary results

**DOI:** 10.1186/s41747-026-00782-3

**Published:** 2026-08-18

**Authors:** Hao Zhang, Yekuan Shi, Yanqin Yu, Zongxi He, Fei Luo, Yue Wu, Tielong Tang, Daiyuan Ma, Suping Li

**Affiliations:** 1 Department of Nuclear Medicine, Affiliated Hospital of North Sichuan Medical College, Nanchong, China; 2 Nuclear Medicine and Radiation Safety Key Laboratory of Sichuan Province, North Sichuan Medical College, Nanchong, China; 3 Medical Imaging Key Laboratory of Sichuan Province, North Sichuan Medical College, Nanchong, China; 4 Department of Urology, Affiliated Hospital of North Sichuan Medical College, Nanchong, China; 5 Department of Oncology, Affiliated Hospital of North Sichuan Medical College, Nanchong, China

**Keywords:** Actinium-225, Molecular targeted therapy, Prostatic neoplasms (castration-resistant), Prostate-specific membrane antigen, Radioimmunotherapy.

## Abstract

**Objective:**

Metastatic castration-resistant prostate cancer (mCRPC) remains associated with poor long-term outcomes, and survival under current treatment is modest. Prostate-specific membrane antigen (PSMA)-targeted alpha therapy using ^225^Ac exhibits superior cytotoxicity compared with beta-emitting radiopharmaceuticals; however, dose-limiting xerostomia from salivary gland accumulation restricts clinical application. ^225^Ac-PSMA-CY313 is a next-generation radioligand designed to reduce off-target salivary uptake while maintaining tumor-targeting efficacy. We evaluated the safety and preliminary efficacy of ^225^Ac-PSMA-CY313 in heavily pretreated mCRPC patients.

**Materials and methods:**

This prospective, open-label, single-arm study enrolled 16 patients with progressive mCRPC refractory to standard therapies. Patients received ^225^Ac-PSMA-CY313 (200 μCi intravenously every 8 weeks). The primary endpoint was treatment-related toxicity per NCI-CTCAE v5.0. Secondary endpoints included ≥ 50% PSA decline (PSA50) and radiographic response per RECIST 1.1/PCWG3.

**Results:**

Xerostomia occurred in 75% of patients, predominantly grade-1 (68.8%), with one grade-2 case. Hematologic toxicities were mild-to-moderate: anemia 68.8% (grade 3: 6.3%), leukopenia 25.0% (all grade 1–2). No grade 4 toxicities or treatment-related deaths occurred. Significant laboratory changes included hemoglobin  decrease (117.7 ± 19.2 *versus* 105.0 ± 19.7 g/L, *p* = 0 .012). PSA50 response was 37.5% (95% confidence interval [CI]: 15.2%–64.6%), with a median PSA change of 18.1% (range: -143.0% to 100.0%). Radiographic response was 31.3% (95% CI: 11.0–58.7%), and the disease control (partial response + stable disease) rate was 62.5% (95% CI: 35.4–84.8%).

**Conclusion:**

In this first-in-human study of 16 patients, ^225^Ac-PSMA-CY313 showed a manageable safety profile with predominantly low-grade xerostomia, a PSA50 response of 37.5%, and an image-based response of 31.3%. Conclusions regarding efficacy and comparative safety require validation in larger prospective trials.

**Clinical trial registration:**

Chinese Clinical Trial Registry: ChiCTR2400083275, Registered 19 April 2024. https://www.chictr.org.cn.

**Key Points:**

- **Question** What are the safety and efficacy outcomes of the novel alpha-emitting radiopharmaceutical ^225^Ac-PSMA-CY313 in advanced mCRPC?
- **Findings** This first-in-human study demonstrates that ²²⁵Ac-PSMA-CY313 produces high PSA response rates with predominantly mild to moderate reversible adverse events.
- **Relevance statement** ^225^Ac-PSMA-CY313 showed promise as a next-generation PSMA-targeted α-therapy with a favorable safety-efficacy profile for advanced prostate cancer patients with limited therapeutic options, supporting its further clinical development.

**Graphical Abstract:**

**Figure:**
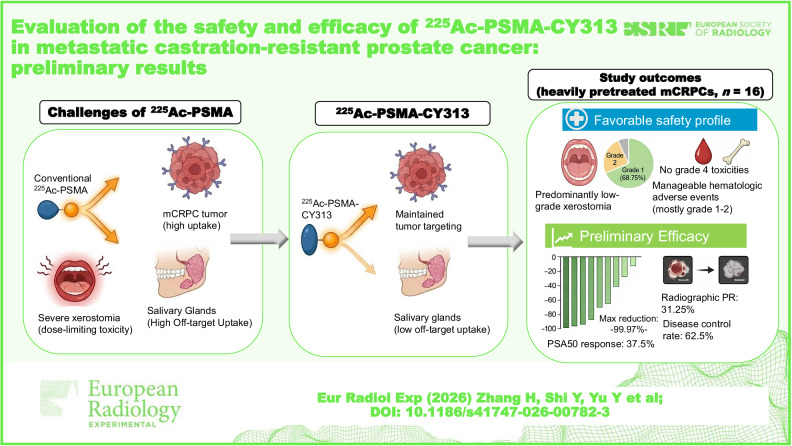

## Background

Metastatic castration-resistant prostate cancer (mCRPC) represents the terminal stage of disease progression, characterized by continued tumor growth despite castrate levels of testosterone. Cross-resistance among androgen receptor signaling inhibitors further limits therapeutic effectiveness [1]. Despite advances in systemic treatment, median overall survival remains approximately 25.6 months [2]. Cytotoxic chemotherapy offers only modest survival benefits with substantial toxicity [3, 4]. These limitations underscore the urgent need for mechanistically distinct therapeutic strategies in mCRPC.

Prostate-specific membrane antigen (PSMA) has emerged as a central molecular target for precision oncology strategies in advanced prostate cancer [5]. PSMA is overexpressed in prostate cancer cells, with intensity correlating with aggressive disease features including high Gleason scores and castration-resistant status [6, 7]. Approximately 90–95% of prostate cancers demonstrate at least focal PSMA expression [8]. Paradoxically, androgen deprivation therapy (ADT) upregulates PSMA expression via activation of the FOLH1 gene enhancer [6]. The therapeutic attractiveness of PSMA lies in its rapid internalization kinetics through clathrin-mediated endocytosis, enabling efficient intracellular accumulation of therapeutic payloads [5]. Despite heterogeneity, most metastatic lesions retain detectable PSMA expression, forming the biological basis for PSMA-targeted radioligand therapy.

The transition from β-particle to α-particle radioligand therapy represents a paradigm shift in therapeutic potency. ^177^Lu-PSMA-617 achieved regulatory approval following the VISION trial [9]. However, β-particles’ long tissue penetration range (approximately 2–10 mm) results in collateral irradiation, and their low linear energy transfer (LET) induces predominantly single-strand DNA breaks. Nearly one-third of patients do not achieve clinically meaningful prostate-specific antigen (PSA) decline [10]. In contrast, ^225^Ac emits high-LET α-particles (80–100 keV/μm) that are 400–500 times more densely ionizing, generating irreparable clustered DNA double-strand breaks. Only 1–3 α-particle traversals induce cell death, compared with the hundreds for β-particles [11]. The short path length (47–85 μm) theoretically limits bystander effects. Preclinical studies demonstrated 50–100-fold greater cytotoxicity than ^177^Lu-PSMA-617 [11]. Additionally, α-particle irradiation may augment antitumor immunity [12].

Clinical application of ^225^Ac-PSMA-617 has produced encouraging responses in heavily pretreated patients, including β-particle refractory therapy. Nevertheless, dose-limiting toxicities constrain widespread adoption. Xerostomia represents the principal limitation, arising from physiological PSMA expression in salivary glands [13]. Severe xerostomia occurs in approximately 10–15% of patients [14] and frequently necessitates dose reduction or discontinuation. These challenges have driven the development of next-generation PSMA-targeted radioligands incorporating structural refinements to improve biodistribution, demonstrating reduced salivary gland uptake while preserving tumor affinity in preclinical studies [11].

^225^Ac-PSMA-CY313 (formerly PSMA-Q) is a novel PSMA-targeting ligand with favorable preclinical properties: high hydrophilicity (log *P* = −3.7 ± 0.4), strong PSMA binding affinity (Ki = 17.1 ± 1.1 nM), and superior tumor-to-muscle ratios compared to established tracers [15–17]. However, CY313 lacks clinical validation and systematic human safety data. This first-in-human study evaluates the safety and preliminary efficacy of ^225^Ac-PSMA-CY313 in mCRPC patients who have exhausted conventional options. Primary objectives assess treatment-emergent adverse events, while secondary endpoints include biochemical responses and radiographic responses. By establishing the safety profile and initial efficacy signals of ^225^Ac-PSMA-CY313, this study provides critical data for the clinical development of this novel α-particle radioligand therapy and informs the design of future dose-optimization and expansion studies in mCRPC.

## Methods

### Study design

**Fig. 1:**
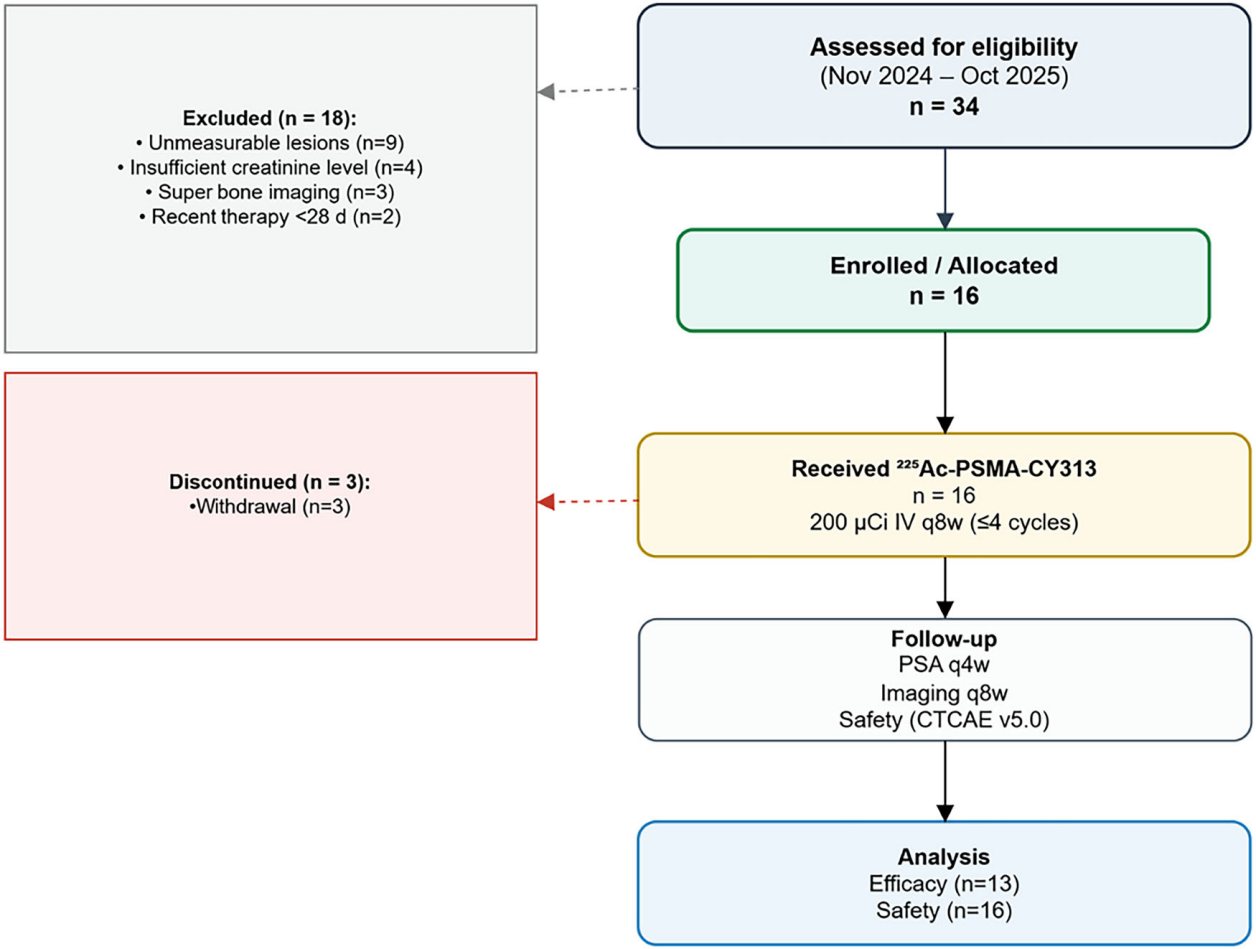
Patient enrollment and treatment flow

This prospective, single-center, single-arm, open-label trial evaluated the safety and preliminary efficacy of ^225^Ac-PSMA-CY313 in patients with mCRPC. The study was conducted at Affiliated Hospital of North Sichuan Medical College between November 20, 2024, and October 30, 2025. The research design and patient screening process are shown in Fig. 1. The study protocol was approved by the institutional review board and ethics committee (approval No. 2024ERJ89-1; March 22, 2024). All study procedures were performed in accordance with the Declaration of Helsinki and Good Clinical Practice guidelines. Written informed consent was obtained from all patients prior to enrollment. The study was registered in the Chinese Clinical Trial Registry (ChiCTR) (No. ChiCTR2400083275, registered on 19 April 2024).

### Patient selection

#### Inclusion criteria

Eligible patients were men aged ≥ 18 years with histologically confirmed prostate adenocarcinoma and documented mCRPC. Key inclusion criteria were as follows: an Eastern Cooperative Oncology Group (ECOG) performance status of 0–2; castrate serum testosterone levels (< 1.7 nmol/L or < 50 ng/dL); at least one measurable metastatic lesion on baseline imaging; positive ^18^F-PSMA-1007 PET/CT with lesion maximum standardized uptake value (SUVmax) exceeding liver SUVmax; and adequate organ function, defined as a white blood cell count ≥ 2.5 × 10^9^/L, neutrophil count ≥ 1.5 × 10^9^/L, platelet count ≥ 100 × 10^9^/L, hemoglobin ≥ 80 g/L, total bilirubin ≤ 1.5 × the upper limit of normal, alanine aminotransferase and aspartate aminotransferase ≤ 3.0 × upper limit of normal, creatinine clearance ≥ 50 mL/min (calculated using the Cockcroft–Gault formula), and serum albumin ≥ 30 g/L.

#### Exclusion criteria

Exclusion criteria were as follows: pathological evidence of sarcomatoid or neuroendocrine differentiation involving > 10% of tumor cells; receipt of systemic anticancer therapy, radiotherapy, or major surgery within 28 days or five drug half-lives (whichever was longer) prior to study drug administration; a superscan pattern on bone scintigraphy; symptomatic spinal cord compression or impending compression; urinary tract obstruction; central nervous system metastases requiring corticosteroid treatment; an estimated life expectancy of less than 6 months; severe cardiac disease, defined as New York Heart Association (NYHA) class III–IV heart failure; active hepatitis B or C infection; and known hypersensitivity to any component of the study drug.

### Treatment protocol

#### Baseline assessment

All patients underwent a comprehensive baseline evaluation within 28 days prior to treatment initiation. Assessments included a complete medical history, physical examination, evaluation of ECOG performance status, complete blood count, comprehensive metabolic panel, measurement of PSA, and a 12-lead electrocardiogram. Baseline imaging comprised contrast-enhanced computed tomography (CT) or magnetic resonance imaging (MRI) of the chest, abdomen, and pelvis, whole-body bone scintigraphy, and ^18^F-PSMA-1007 PET/CT.

#### ^225^Ac-PSMA-CY313 administration

^225^Ac-PSMA-CY313 was administered intravenously at a starting dose of 200 μCi (±10%) per cycle, with protocol-defined dose modifications permitted for toxicity management. The radiopharmaceutical was diluted in normal saline and delivered via a microinfusion pump over approximately 10 min, followed by a saline flush. Treatment consisted of up to four cycles administered at 8-week intervals (± 1 week). Dose reductions to 150 μCi were allowed for grade 3 hematologic toxicity persisting > 2 weeks or any grade 4 toxicity. Treatment discontinuation criteria included: grade 4 hematologic toxicity lasting > 7 days, grade ≥ 3 non-hematologic toxicity (except nausea/vomiting) not resolving to grade ≤ 1 within 4 weeks, or any life-threatening adverse event. All patients continued ADT throughout the study to maintain castrate serum testosterone levels.

#### Follow-up schedule

Safety assessments were conducted at scheduled visits and included physical examination, vital signs, ECOG performance status, laboratory tests (complete blood count, comprehensive metabolic panel, and PSA), and adverse event monitoring. PSA measurements were performed every 4 weeks (± 3 days) from cycle 1 day 1 until disease progression or data cutoff. Imaging assessments, including CT or MRI and bone scintigraphy, were performed every 8 weeks (± 1 week) to evaluate treatment efficacy, continuing until disease progression or data cutoff.

### Safety assessment

#### Adverse events monitoring

All adverse events occurring from the time of informed consent through 8 weeks after the last dose were recorded and graded according to the National Cancer Institute Common Terminology Criteria for Adverse Events, version 5.0 (NCI-CTCAE v5.0). Investigators assessed the relationship between adverse events and study treatment using predefined causality categories: definitely related, probably related, possibly related, unlikely related, or definitely not related. Events classified as definitely, probably, or possibly related were considered treatment-related adverse events. Serious adverse events were defined as events resulting in death, life-threatening situations, hospitalization or prolongation of hospitalization, persistent disability, or other medically important conditions.

#### Laboratory evaluations

Laboratory assessments were performed at baseline, prior to each treatment cycle, and during follow-up visits. These included complete blood count with differential, comprehensive metabolic panel (including liver and kidney function tests), and PSA levels. Hematologic toxicity was closely monitored due to the potential for bone marrow suppression associated with α-particle therapy. Laboratory abnormalities were graded according to NCI-CTCAE v5.0 criteria.

### Efficacy assessment

#### Biochemical response (PSA)

PSA response was assessed according to Prostate Cancer Clinical Trials Working Group 3 (PCWG3) criteria. A PSA response was defined as a ≥ 50% decline from baseline, confirmed by a second measurement at least 3 weeks later. PSA progression was defined as an increase of ≥ 25% and an absolute rise of ≥ 2 ng/mL from the baseline or nadir value (whichever was lower), confirmed by a second measurement at least 3 weeks later.

#### Image-based response evaluation

Image-based response was assessed using Response Evaluation Criteria in Solid Tumors version 1.1 (RECIST 1.1) for soft tissue lesions and PCWG3 criteria for bone lesions. Imaging assessments were performed every 8 weeks until confirmed disease progression or data cutoff. The radiographic response rate was defined as the proportion of patients achieving a best response of complete response (CR) or partial response (PR).

### Statistical analysis

Given the exploratory nature of this first-in-human study, a formal sample size calculation was not performed. A total of 16 patients were enrolled. To characterize the safety profile and generate preliminary efficacy signals for ^225^Ac-PSMA-CY313. Descriptive statistics were used to summarize patient characteristics, safety outcomes, and treatment responses. Continuous variables were reported as mean ± standard deviation or median with range, and categorical variables were expressed as frequencies and percentages. Patients without evaluable radiographic assessments due to early discontinuation or absence of measurable disease (*n* = 3) were excluded from radiographic response calculations but included in safety and PSA analyses. No data imputation was performed. PSA response rates and the radiographic response were calculated as proportions. All statistical analyses were performed using SPSS version 25.0.

### Large language models usage statement

During the preparation of this work, the authors used ChatGPT4.0 in order to improve the readability and language of the manuscript. After using this tool, the authors reviewed and edited the content as needed and take full responsibility for the content of the publication.

## Results

### Patient characteristics

Between November 20, 2024, and October 30, 2025, a total of 16 patients with mCRPC were enrolled and received at least one dose of ^225^Ac-PSMA-CY313. Baseline characteristics are summarized in Table 1. The median age was 68 years (range, 54–78). Most patients (75%, *n* = 12) had an ECOG performance status of 0, 18.8% (*n* = 3) had ECOG 1, and 6.3% (*n* = 1) had ECOG 2. Regarding PSMA PET inclusion criteria, the median baseline SUVmax of target lesions was 18.7 (range: 8.3–47.2), with a median liver SUVmax of 5.1 (range: 3.8–6.9).

**Table 1:** Patient characteristics

Characteristics	Value
Number of patients (*n*)	16
Median age (years)	68
ECOG Score, *n* (%)	
0	12 (75.00%)
1	3 (18.75%)
2	1 (6.25%)
Sites of metastases, *n* (%)	
Bone	49 (66.22%)
Lymph node	18 (24.32%)
Other organs	7 (9.45%)
Previous therapies, *n* (%)	
Local therapy	
Radical prostatectomy/prostatectomy	6 (37.50%)
Palliative radiotherapy (to bone/lymph nodes)	4 (25.00%)
Other local therapy (*e.g.*, TURP)	2 (12.50%)
Systemic therapy	
Endocrine therapy	
Any traditional ADT (bilateral orchiectomy or GnRH agonists)	12 (75.00%)
Bilateral orchiectomy	4 (25.00%)
GnRH agonists (goserelin/leuprorelin)	8 (50.00%)
Any first-generation antiandrogen (bicalutamide/flutamide)	11 (68.75%)
Any novel hormonal agent (abiraterone acetate/enzalutamide/apalutamide/darolutamide/rezvilutamide)	15 (93.75%)
≥ 2 Novel hormonal agents (abiraterone acetate/enzalutamide/apalutamide/darolutamide/rezvilutamide)	5 (31.25%)
Chemotherapy (docetaxel)	9 (56.25%)
Targeted therapy	
PARP Inhibitor (Olaparib)	2 (12.50%)
Other investigational agents^*^ (TQB3823, TQB392, CBP-1018, Mevrometostat)	1 (6.25%)
Other systemic agent (Denosumab^**^)	2 (12.50%)

*ADT* Androgen deprivation therapy, *ECOG* Eastern Cooperative Oncology Group, *GnRH* Gonadotropin-releasing hormone

^*^ Other investigational agents: includes experimental drugs with unspecified mechanisms from clinical trial records

^**^ Denosumab: a RANK ligand inhibitor used for bone health management. It is listed here as a relevant supportive care agent

The cohort was heavily pretreated, with all patients having received multiple lines of systemic therapy. All 16 patients (100%) had previously undergone ADT, with 75% (*n* = 12) receiving traditional ADT (bilateral orchiectomy in 25% and GnRH agonists in 50%) and 68.8% (*n* = 11) receiving first-generation antiandrogens. Notably, 93.8% (*n* = 15) had been treated with at least one novel hormonal agent (NHA), including abiraterone acetate, enzalutamide, apalutamide, darolutamide, or rezvilutamide, and 31.3% (*n* = 5) had received ≥ 2 NHAs sequentially. Prior chemotherapy included docetaxel in 56.3% (*n* = 9) of patients; the relatively lower exposure reflects contemporary treatment patterns in which NHAs are increasingly administered before chemotherapy, and some patients are ineligible for chemotherapy due to declining performance status or comorbidities. Additionally, 37.5% (*n* = 6) had undergone radical prostatectomy, 25% (*n* = 4) had received palliative radiotherapy to bone or lymph node metastases, and 12.5% (*n* = 2) had received investigational agents, indicating that standard treatment options had been largely exhausted.

Treatment exposure and follow-up: the median number of treatment cycles was 4 (range: 1–4), with 9 patients (56.3%) completing all planned cycles. Early discontinuation reasons included disease progression (*n* = 3) and patient preference (*n* = 4). No dose modifications were required, with all patients receiving 200 μCi per administered cycle. The median follow-up duration was 253.5 days (range: 54–534 days).

### Safety profile

^225^Ac-PSMA-CY313 demonstrated a manageable safety profile in all 16 treated patients. Treatment-related adverse events are summarized in Table 2. The most frequently observed toxicity was xerostomia, occurring in 75% of patients (*n* = 12), Most cases were grade 1 (68.8%, *n* = 11), with only one patient (6.3%) experiencing grade 2. Xerostomia predominantly manifested after the first treatment cycle (200 μCi) in 10/12 affected patients, with 2 patients developing symptoms after cycle 2. Once established, xerostomia persisted through the end of follow-up in all cases, though severity remained stable (grade 1) in most patients without progression. Hematologic toxicities were generally mild to moderate. Anemia was observed in 68.8% of patients (*n* = 11); with 37.5% grade 1, 25.0% grade 2, and 6.3% grade 3. Leukopenia occurred in 25% of patients (*n* = 4), evenly split between grade 1 and grade 2. No grade 4 hematologic toxicities were reported.

**Table 2:** Therapy-related side effects after ^225^Ac -PSMA-CY313 according to NCI-CTCAE v5.0. (*n* = 16)

	Grade I	Grade II	Grade III	Grade IV	Note
Anemia	6	4	1	0	G3 event at C1D28; persisted for 6 weeks, managed with erythropoietin support, resolved to G2, assessed as probably related to study drug
Xerostomia	11	1	0	0	
Fatigue	3	3	0	0	
Nausea	1	1	1	0	G3 event at C1D4; lasted 4 days, managed with antiemetics, resolved completely, assessed as possibly related
Dyspepsia	1	3	0	0	
Diarrhea	1	2	0	0	
Anorexia	1	2	0	0	
Leukopenia	2	2	0	0	
Hypokalemia	2	1	1	0	G3 event at C4D1; corrected within 48 h with IV potassium supplementation, assessed as possibly related
Vomiting	0	2	0	0	
Weight Decreased	1	1	0	0	
Hyponatremia	2	0	0	0	
Hypocalcemia	0	1	0	0	
Hypoalbuminemia	1	0	0	0	
Hepatic Function Abnormal	1	0	0	0	

*NCI-CTCAE* National cancer institute common terminology criteria for adverse events

Gastrointestinal adverse events were common but predominantly low-grade. Fatigue affected 37.5% of patients (*n* = 6), dyspepsia 25% (*n* = 4), diarrhea 18.8% (*n* = 3), nausea 18.8% (*n* = 3), and anorexia 18.8% (*n* = 3). Only one patient experienced grade 3 nausea; all other gastrointestinal events were grade 1–2. Electrolyte disturbances included hypokalemia in 25% of patients (*n* = 4, including one grade 3 event), hyponatremia in 12.5% (*n* = 2, all grade 1), and hypocalcemia in 6.3% (*n* = 1, grade 2).

Changes in laboratory parameters from baseline to post-treatment are summarized in Table 3. Statistically significant decreases were observed in red blood cell count (4.18 ± 0.79 *versus* 3.72 ± 0.82 × 10¹²/L, *p* = 0.018, Cohen’s *d* = 0.57), hemoglobin (117.7 ± 19.2 *versus* 105.0 ± 19.7 g/L, *p* = 0.012, Cohen’s *d* = 0.65), and albumin levels (44.0 ± 4.9 *versus* 41.5 ± 5.3 g/L, *p* = 0.018, Cohen’s *d* = 0.49). A significant increase in gamma-glutamyl transferase (GGT) was also noted (53.2 ± 84.7 *versus* 200.4 ± 533.5 U/L, *p* = 0.028, Cohen’s *d* = 0.39), although this elevation displayed substantial interpatient variability. White blood cell count (Cohen’s *d* = 0.14), platelet count (*p* = 0.058, Cohen’s *d* = 0.51), and renal function parameters (creatinine: Cohen’s *d* = 0.15) showed no statistically significant changes. No treatment-related deaths or discontinuations due to toxicity occurred during the study.

**Table 3:** Laboratory parameters at baseline and after ^225^Ac-PSMA-CY313 (*n* = 16)

Parameter	Baseline(mean ± SD)	After last dose ^225^Ac-PSMA-CY313 (mean ± SD)	Mean change	*p*-value	Cohens’ *d*
WBC (×10⁹/L)	7.08 ± 2.47	6.59 ± 4.30	-0.49	0.68	0.14
RBC (×10¹²/L)	4.18 ± 0.79	3.72 ± 0.82	-0.46	0.018	0.57
HGB (g/L)	117.7 ± 19.2	105.0 ± 19.7	-12.7	0.012	0.65
PLT (×10⁹/L)	232.1 ± 78.9	190.4 ± 83.1	-41.7	0.058	0.51
ALT (U/L)	22.9 ± 18.4	19.6 ± 16.1	-3.3	0.54	0.19
AST (U/L)	29.7 ± 19.1	33.7 ± 21.6	4.0	0.47	0.20
ALP (U/L)	151.6 ± 74.5	203.0 ± 250.0	51.4	0.09	0.28
GGT (U/L)	53.2 ± 84.7	200.4 ± 533.5	147.2	0.028	0.39
Cr (µmol/L)	68.3 ± 17.2	72.5 ± 35.8	4.2	0.49	0.15
ALB (g/L)	44.0 ± 4.9	41.5 ± 5.3	-2.5	0.018	0.49

*ALB* Albumin, *ALP* Alkaline phosphatase, *ALT* Alanine aminotransferase, *AST* Aspartate aminotransferase, *Cr* Creatinine, *GGT*,γ‑glutamyl transferase, *HGB* Hemoglobin, *PLT* Platelet, *RBC* Red blood cell, *WBC* White blood cell

### Treatment efficacy

#### Biochemical response

**Fig. 2:**
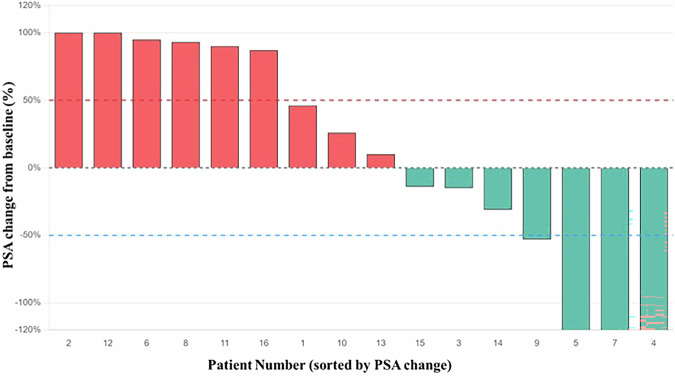
Waterfall plot demonstrating percentage change in serum PSA levels from baseline following ^225^Ac-PSMA-CY313 therapy (*n* = 16). The vertical axis represents the percentage change in PSA calculated as (baseline PSA - post-treatment PSA)/baseline PSA × 100%. Patients are arranged along the horizontal axis sorted by magnitude of PSA change. Red bars indicate patients with PSA decline from baseline, while green bars represent patients with PSA increase. The horizontal dashed line at 50% marks the threshold for PSA50 response

PSA responses following ^225^Ac-PSMA-CY313 therapy are summarized in Table 4 and illustrated in Fig. 2. Among the 16 treated patients, six (37.5%; 95% CI: 15.2%–64.6%) achieved a ≥ 50% decline in PSA from baseline (PSA50 response). The median PSA change from baseline was 18.13% (range: -143.0% to 100.0%). At data cutoff, the median PSA level had decreased from 152.45 ng/mL at baseline to 104.57 ng/mL (range: 0.04–7,650 ng/mL). The waterfall plot (Fig. 2) illustrates the heterogeneous PSA responses observed across the cohort. Nine patients (56.3%) experienced some degree of PSA decline, although the magnitude of response varied widely. Notably, three patients achieved PSA reductions exceeding 90%, including one patient with a remarkable 99.97% decrease. Conversely, five patients (31.3%) experienced PSA progression during treatment.

**Table 4:** PSA response and overall radiographic response assessment following ^225^Ac-PSMA-CY313 therapy (*n* = 16)

Parameter	Value
Number of patients	16
Baseline PSA (ng/mL), median (range)	152.45 (2.64–5,000)
Last‑follow‑up PSA (ng/mL), median (range)	104.57 (0.04–7,650)
PSA change from baseline (%), median (range)	18.13 (-143.00 to 99.97)
Patients with ≥ 50% PSA decline, *n* (%)	6 (37.5%)
Overall response^*^, *n* (%)	
PR	5 (31.25%)
SD	5 (31.25%)
PD	3 (18.75%)
NA	3 (18.75%)

*NA* Not applicable, *PCWG3* Prostate cancer clinical trials working group 3, *PD* Progressive disease, *PR* Partial response, *PSA* Prostate specific antigen, *RECIST* Response evaluation criteria in solid tumors, *SD* Stable disease

* Overall response was assessed according to RECIST 1.1 criteria combined with PCWG3 guidelines for lesions

#### Image-based response

Radiographic responses were assessed using RECIST 1.1 criteria for soft tissue lesions and PCWG3 guidelines for bone lesions (Table 4). Among the 16 patients, PR was observed in 5 patients (31.3%), stable disease (SD) in 5 patients (31.3%), and progressive disease (PD) in 3 patients (18.8%). Three patients (18.8%) were not evaluable for radiographic response due to early treatment discontinuation or absence of measurable disease at baseline. The image-based response was 31.3% (95% CI: 11.0%–58.7%), and the disease control rate (PR + SD) was 62.5% (95% CI: 35.4%–84.8%). The correlation between PSA and radiographic responses was heterogeneous. Of the five patients achieving PR, two had concurrent PSA50 responses, whereas three demonstrated radiographic improvement despite < 50% PSA decline. Conversely, four patients with PSA50 responses did not achieve radiographic PR, exhibiting SD instead.

### Representative case illustration

**Fig. 3:**
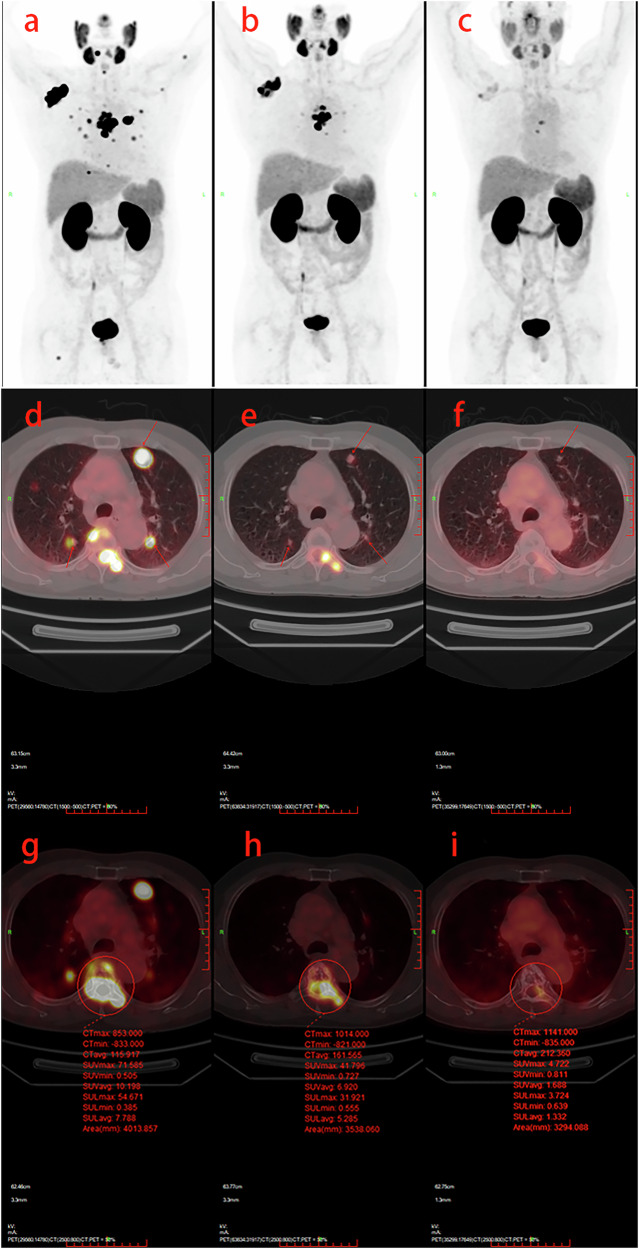
Representative ^18^F-PSMA-1007 PET/CT imaging demonstrating treatment response to ^225^Ac-PSMA-CY313 therapy. Images show from a representative patient at baseline (**a**, **d**, **g**), after one cycle (**b**, **e**, **h**), and after four cycles (**c**, **f**, **i**) of ^225^Ac-PSMA-CY313 treatment. The area indicated by the red arrow and within the red circle represents the lesion expressing PSMA

Figure 3 depicts a representative case demonstrating an exceptional response to ^225^Ac-PSMA-CY313 therapy. The patient, a 61-year-old man with heavily pretreated mCRPC, presented with extensive osseous and pulmonary metastases at baseline. Pre-treatment ^18^F-PSMA-1007 PET/CT imaging (Fig. 3a, d, g) revealed high PSMA expression across multiple bone lesions in both the axial and appendicular skeleton, as well as bilateral pulmonary metastases with intense PSMA uptake.

Following one cycle of ^225^Ac-PSMA-CY313 (200 μCi), imaging (Fig. 3b, e, h) demonstrated marked reductions in both lesion number and metabolic activity. After four treatment cycles, the patient achieved a remarkable biochemical and radiographic response. Serum PSA decreased from 157.9 ng/mL at baseline to 0.28 ng/mL, representing a 99.97% reduction. Post-treatment ^18^F-PSMA-1007 PET/CT (Fig. 3c, f, i) showed substantial reductions in size and metabolic activity of pulmonary metastases and a marked decrease in the extent and metabolic activity of osseous lesions.

Clinically, the patient experienced significant symptomatic improvement, with complete resolution of baseline back and chest pain that had previously required regular analgesics. Treatment was well tolerated, with only grade 1 xerostomia and grade 1 fatigue reported. This case exemplifies the potential for profound and durable responses to ^225^Ac-PSMA-CY313 in selected patients with mCRPC, even in the context of high disease burden and extensive prior therapy.

## Discussion

The safety profile of ^225^Ac-PSMA-CY313 in this initial clinical evaluation appears notably improved, particularly with respect to xerostomia, the most common dose-limiting toxicity associated with alpha-particle therapy in this setting. A meta-analysis of ^225^Ac-PSMA-617 demonstrated that xerostomia of any degree occurred in 77.1% of patients, with grade III xerostomia in 3.0% [18]. In contrast, our cohort showed 75% overall xerostomia with only one grade 2 and no grade 3 or higher events.

The reduced salivary toxicity observed with CY313 is likely attributable to molecular structural optimizations, including optimized linker design and chelator structure conferring higher aqueous solubility, which may reduce nonspecific binding in salivary tissue [19, 20]. Dosimetric analysis published separately demonstrated favorable biodistribution with a salivary gland absorbed dose of 0.50 ± 0.47 Gy, supporting the mechanistic basis for reduced xerostomia severity observed clinically [21]. Clinically, this improved safety profile is meaningful, as salivary gland dysfunction substantially impairs quality of life and frequently necessitates treatment discontinuation. In our study, 68.8% of patients experienced only grade 1 xerostomia—an encouraging result compared with prior reports, where grade 1 xerostomia occurred in 10/18 patients and grade 2 in 2/18 patients after one cycle of Tandem-PRLT [22]. Nonetheless, the relatively short median follow-up warrants longer observation to fully assess the risk of delayed salivary gland dysfunction.

Importantly, regarding xerostomia duration, most cases (11/12, 91.7%) persisted throughout the follow-up period without complete resolution by the data cutoff. This finding highlights a critical limitation of our current analysis: while the severity of xerostomia appears reduced compared to other alpha-emitting radioligands, the persistence of symptoms suggests that the long-term burden may be underestimated. Long-term follow-up beyond 6 months is ongoing to fully characterize the persistence and potential reversibility of xerostomia. Furthermore, future studies will incorporate validated patient-reported outcome measures such as the Xerostomia Inventory to better quantify the clinical impact on patients’ daily functioning and quality of life. These tools will provide more nuanced assessment beyond CTCAE grading and help establish whether the apparent reduction in xerostomia severity translates into meaningful clinical benefit for patients.

The hematologic safety profile of ^225^Ac-PSMA-CY313 shows favorable tolerability, with only 6.25% of patients experiencing grade 3 anemia and no grade 4 hematologic toxicities observed. Sathekge et al's real-world data showed approximately 15% of patients experiencing anemia with ^225^Ac-PSMA-617, though no other grade 3 or 4 hematotoxicities were noted [23]. Clinical data show that hematologic toxicity with ^225^Ac-PSMA-617 particularly occurs in patients with preexisting hematologic impairment, prior myelotoxic therapies, and high bone tumor burden [24].

Our dosing regimen of 200 μCi every 8 weeks likely contributed to the favorable hematologic profile by allowing adequate marrow recovery between cycles. Notably, 66.22% of our patients had bone metastases yet maintained excellent hematologic tolerance, suggesting CY313 may offer an improved therapeutic window. Statistically significant but clinically manageable decreases in hemoglobin (117.7 ± 19.2 *versus* 105.0 ± 19.7 g/L, *p* = 0.012) and red blood cell counts remained within acceptable ranges for most patients. This controlled hematologic impact, together with the absence of treatment discontinuations due to cytopenias, supports the feasibility of CY313 administration in this heavily pretreated population.

The therapeutic efficacy of ^225^Ac-PSMA-CY313 in this extensively pretreated cohort provides encouraging preliminary signals. Despite this extensive pretreatment, 37.5% (95% CI: 15.20%–64.60%) achieved PSA50 response, and 31.3% (95% CI: 10.99%–58.66%) demonstrated radiographic PR. However, given the small sample size, wide confidence intervals, and limited follow-up duration, these preliminary findings require cautious interpretation. The TheraP trial showed 66% PSA50 response with ^177^Lu-PSMA-617 [25], while meta-analysis of ^225^Ac-PSMA-617 studies reported pooled PSA50 rates of 66.1% [18]. While direct comparisons are inappropriate given the differences in study design, patient populations, and sample sizes, our observed response rates should be contextualized within the wide confidence intervals that reflect the uncertainty inherent in this small cohort. The disease control rate of 62.5% suggests meaningful clinical activity. Notably, one patient achieved 99.97% PSA reduction, exemplifying potential for profound responses in selected cases. However, 37.5% experiencing progression indicates inherent resistant subpopulations. These findings, while encouraging in this challenging population, require validation in larger cohorts to determine optimal patient selection and establish CY313’s position relative to existing radioligand therapies.

The observed discordance between biochemical and radiographic responses—37.5% achieving PSA50 response but only 31.3% showing radiographic PR—reflects complex biological phenomena inherent to radioligand therapy. Specifically, among the 5 patients who achieved radiographic PR, only 2 (40%) achieved PSA50 response, highlighting the dissociation between these response metrics. This discordance, reported in up to 24.5% of cases [26], may reflect several mechanisms: differential PSMA expression heterogeneity across metastatic sites, treatment-induced phenotypic changes including neuroendocrine differentiation, α-particle immunomodulatory effects, potential selection of PSMA-negative clones post-treatment, or limitations of conventional response criteria in evaluating radioligand therapy effects [27]. The 25% of patients with SD despite biochemical response in our cohort may reflect these biological complexities. The prognostic significance of PSA dynamics remains relevant, as sustained PSA decreases correlate with improved survival [28]. But these findings underscore the limitations of single biomarker assessment and support integrated evaluation approaches such as RECIP 1.0 for comprehensive response assessment [29], particularly as conventional PCWG3 criteria were developed for traditional therapies rather than radioligand treatments. Future phase 2 studies will incorporate serial PSMA PET imaging to quantify SUVmax changes and correlate with treatment response. Progression-free and overall survival will be formally assessed as secondary endpoints with adequate follow-up. These comprehensive assessments, combined with multi-parametric response evaluation including circulating tumor DNA and patient-reported outcomes, will better capture the multifaceted effects of alpha radioligand therapy.

This preliminary investigation has several inherent limitations. Key limitations include: (1) small sample size (*n* = 16) limiting comprehensive safety profile characterization and efficacy assessment, particularly for detecting rare adverse events; (2) insufficient follow-up duration to assess long-term toxicities or survival outcomes; (3) single-arm design precluding direct comparison with standard treatments or other ^225^Ac-PSMA ligands; and (4) fixed 200 μCi dose without exploration of dose-response relationships.

To address these limitations and establish CY313’s clinical role, systematic future investigations are essential. Phase 2 dose-optimization studies with larger patient numbers (*n* = 60–80) and extended follow-up are currently being planned to validate these preliminary observations and determine the optimal dosing strategy. Head-to-head comparisons with existing therapies, particularly ^177^Lu-PSMA-617 and ^225^Ac-PSMA-617, will be crucial for establishing CY313’s position in the treatment sequence. While definitive conclusions regarding therapeutic superiority cannot be drawn from this initial cohort, these planned studies will provide the evidence necessary to guide clinical decision-making in mCRPC management.

This first-in-human exploratory study provides initial safety and efficacy data for ^225^Ac-PSMA-CY313 in 16 heavily pretreated mCRPC patients. The observed safety profile, with predominantly grade 1 xerostomia and limited hematologic toxicity, appears manageable. Preliminary efficacy signals include PSA50 response of 37.5% (95% CI: 15.20%–64.60%) and radiographic response of 31.25% (95% CI: 10.99%–58.66%) in this challenging patient population. However, given the small sample size, wide confidence intervals, and limited follow-up duration, these preliminary findings require cautious interpretation. While these early results support continued investigation, definitive conclusions regarding the therapeutic advantages of CY313 over existing radioligand therapies await validation in adequately powered, controlled phase 2 trials with longer follow-up. Future studies should focus on dose optimization, patient selection biomarkers, and direct comparative effectiveness evaluation to establish CY313’s clinical positioning in the mCRPC treatment paradigm.

## Data Availability

The datasets generated during and/or analyzed during the current study are available from the corresponding author on reasonable request.

## Abbreviations

ADT: Androgen deprivation therapy
CT: Computed tomography
ECOG: Eastern Cooperative Oncology Group
mCRPC: Metastatic Castration-Resistant Prostate Cancer
NCI-CTCAE: National Cancer Institute Common Terminology Criteria for Adverse Events
NHA: Novel hormonal agents
PCWG3: Prostate cancer clinical trials working group 3
PR: Partial response
PSA: Prostate-specific antigen
PSMA: Prostate-specific membrane antigen
RECIP: Response evaluation criteria in PSMA PET/CT
RECIST: Response evaluation criteria in solid tumors
SD: Stable disease
SUVmax: Maximum standardized uptake value
